# Gender Identity and Questioning in Klinefelter's Syndrome

**DOI:** 10.1192/bjo.2022.176

**Published:** 2022-06-20

**Authors:** Valerie Cai, Tet Yap

**Affiliations:** 1 King's College London, London, United Kingdom; 2 Guy's and St Thomas' NHS Foundation Trust, London, United Kingdom

## Abstract

**Aims:**

KS is a congenital condition with 47, XXY chromosome karyotype. Due to a lack of understanding of the condition amongst healthcare providers, KS is grossly underdiagnosed, with most patients never receiving a diagnosis. Within this population, gender dysphoria is suggested to be of higher incidence than in the general population. To establish the validity of this claim and to improve care for patients with KS, particularly in the area of gender service provision, we need to identify whether there is a significant proportion of XXY individuals that experience gender dysphoria. The aim of our study is to determine whether UK patients with a diagnosis of KS are more predisposed to gender dissatisfaction.

**Methods:**

A PRISMA literature review was conducted on the epidemiology, management, and treatment outcomes of KS patients with gender dysphoria. Based on the results of the literature review, we then conducted a cross-sectional survey of patients serviced by the Klinefelter Syndrome Association on gender satisfaction. The survey recorded 81 responses.

**Results:**

Of the entire study population, gender distribution was 65% male, 6% female, 4% non-binary, 2% gender fluid, 3% neither, 1% equally male and female, and 1% intersex. This contrasted with most patients’ assigned birth on their birth certificate, which was 92.5% male and 3.75% female. Most patients surveyed enjoyed living as the sex written on their birth certificate (61.64%), which seemed to correlate closely with the proportion of patients that identified as male (65%).

**Conclusion:**

Literature Review: As a whole, KS patients documented in research presented to psychiatric and sexual health services during adulthood, requesting either sex reassignment surgery or changes to hormonal replacement therapy. The sparse amount of research over a long period of time has created a reliance on outdated research techniques. Patient Survey: Survey results show that there are a significant proportion of survey respondents that do not identify as male despite it being written on their birth certificate; however, the majority prefer to be identified as male. This suggests that certain individuals with KS are at higher risk of gender dissatisfaction and dysphoria. Importantly, these observations are not substantiated with clinical judgement of a psychiatrist/mental health worker, which should aim to be incorporated in future research. Additionally, longitudinal studies should aim to establish whether certain age groups would be more at risk of gender dissatisfaction and gender dysphoria or if trends change with age.

